# Intestinal failure: a review

**DOI:** 10.12688/f1000research.12493.1

**Published:** 2018-01-18

**Authors:** Philip Allan, Simon Lal

**Affiliations:** 1 Translational Gastroenterology Department, University Hospitals Oxford NHS Foundation Trust, Oxford, UK; 2 Intestinal Failure Unit, Salford Royal Foundation Trust, Salford, UK

**Keywords:** intestinal failure, thromboembolism, home parenteral nutrition

## Abstract

Intestinal failure (IF) is the inability of the gut to absorb necessary water, macronutrients (carbohydrate, protein, and fat), micronutrients, and electrolytes sufficient to sustain life and requiring intravenous supplementation or replacement. Acute IF (types 1 and 2) is the initial phase of the illness and may last for weeks to a few months, and chronic IF (type 3) from months to years. The challenge of caring for patients with IF is not merely the management of the underlying condition leading to IF or the correct provision of appropriate nutrition or both but also the prevention of complications, whether thromboembolic phenomenon (for example, venous occlusion), central venous catheter-related bloodstream infection, IF-associated liver disease, or metabolic bone disease. This review looks at recent questions regarding chronic IF (type 3), its diagnosis and management, the role of the multidisciplinary team, and novel therapies, including hormonal treatment for short bowel syndrome but also surgical options for intestinal lengthening and intestinal transplant.

## Introduction

Intestinal failure (IF) is the inability of the gut to absorb sufficient macronutrients (carbohydrates, protein, and fat), micronutrients (vitamins, minerals, and electrolytes), or water, resulting in the need for intravenous supplementation to maintain health or facilitate growth ^1^. This is a relatively rare condition, affecting about 50 per million people with chronic IF (CIF) (type 3, see below) needing long-term home parenteral nutrition (HPN), but the impact on both the individual and the healthcare economy is high. The length of stay at the time of diagnosis of IF is commonly in the order of weeks to months and typically costs up to USD $150,000 per annum to keep someone on HPN ^2– 9^. This review will look at outcomes, complications, and current treatments for CIF (type 3).

## Intestinal failure aetiology

The cause of IF can be categorised according to timing of presentation (congenital versus acquired, such as gastroschisis versus surgical complications), speed of onset (rapid versus prolonged, such as mesenteric ischaemia versus Crohn’s disease or chronic intestinal pseudo-obstruction), underlying pathology (benign versus malignant), locality (localised to gastrointestinal tract versus systemic disease), and duration (short-term versus long-term). IF is frequently also categorised into three functional subtypes ^10^, as seen in Table 1. Type 1 is usually self-limiting and typical examples include post-operative ileus. Type 2 IF presents following intra-abdominal surgery with metabolically unstable patients often with hostile abdomens, fistulae, or adhesions. These patients require a multidisciplinary team (MDT) approach to their care, aiming to stabilise and move the patient to a position where the team can start repairing the physical and psychological distress encountered. These may include patients presenting following an acute traumatic event such as a traffic accident, following surgical procedures associated with anastomotic leaks, vascular or viscous injury during other surgery, creation of laparostomy (open abdominal wound), and acute unpredictable events such as enterocutaneous fistulae, intestinal volvulus, and mesenteric infarction. Type 3 IF is CIF and includes patients who have progressed from instability to stability, requiring long-term intravenous management of their IF over months to years; it may be reversible or irreversible ^11, 12^. There is a further helpful clinical classification of type 3 based on calories consumed (kcal/kg per day: 0, 1–10, 11–20, >20) and volume of intravenous support required (<1, 1.001–2, 2.001–3, >3 L) ^13^. IF can be further classified according to pathophysiology: short bowel syndrome (SBS), intestinal fistulae, dysmotility, mechanical obstruction, and extensive small bowel mucosal disease.

**Table 1.** Defining intestinal failure subtypes.

Subtype	Presentation timing	Speed of onset	Locality of disease	Pathology	Duration
Type 1	Acquired	Acute	GI and systemic	Benign and malignant	<28 days
Type 2	Congenital/acquired	Acute	GI and systemic	Benign and malignant	Weeks to months
Type 3	Congenital/acquired	Chronic	GI and systemic	Benign and malignant	Months to years

GI, gastrointestinal.

Such classifications enable consistency for both clinical reporting of patients encountered and outcomes across specialist centres. In particular, the pathophysiological classification enables a clear understanding of the underlying mechanism and aetiology of the IF. The clinical classification according to level of calorific/fluid support required enables sensible understanding of disease severity, potentially facilitating a targeted approach to novel therapies aimed at minimising disease impact on the individual.

## Complications of intestinal failure

Though life-sustaining, HPN is associated with complications, including those associated with the central venous catheter (CVC) used to administer the parenteral nutrition (PN), IF-associated liver disease (IFALD), an impact on quality of life (QoL), and metabolic bone problems. In non-cancer IF diagnoses, the survival is remarkable; 1-, 5-, 10-, and 20-year survival has been reported as 93%, 71%, 59%, and 28%, respectively ^14^. Wherever a patient is—whether at home, in a specialist centre, or in a district general hospital—they should expect exceptional care of their CVC. The fragility of their venous access should ensure that all hospitals develop a standard of care to minimise complications. Well-skilled and well-resourced MDTs ^15– 19^ that maintain stability in terms of makeup of healthcare professionals and have clear protocols ^20, 21^ do better than teams experiencing high numbers of staff changes or organisational changes ^22^.

### Diagnosis of catheter-related bloodstream infections

One of the controversial areas within IF currently is the process of actually making the diagnosis of a catheter-related bloodstream infection (CRBSI) despite clear standards laid out by the Centers for Disease Control and Prevention, the Infectious Disease Society of America, and the European Society for Parenteral and Enteral Nutrition (ESPEN) ^23– 25^, whose current recommendations include quantitative and qualitative methods to diagnose a CRBSI. Unfortunately, the standardisation of care both nationally and internationally through obtaining appropriate cultures requires investment in infrastructure and training. Quantitative analysis using pour plates, though the gold standard ^23^, exists in only a limited number of exceptional centres ^21^. Time to positivity needs a clear protocol of matched volumes added to the culture bottles from both peripheral and central access points ^26^ followed by equal handling of the samples and presenting them to the lab within 30 minutes. Not unsurprisingly, within the literature, broad diagnostic criteria are followed and some are pragmatically decided on the basis of there being no other source and improvement in symptoms on treatment ^27^ through to making a clinical diagnosis using any positive blood culture and temperature, but this may result in over-diagnosis and inappropriate antibiotic stewardship in at least 46% ^28^.

Attempts at non-invasive methods of detecting a CRBSI remain challenging. Immunoglobulin levels to flagellin and lipopolysaccharide do not differentiate CRBSI from any other microbial infection ^29^. A non-specifically unwell patient with a newly abnormal C-reactive protein, albumin, or bilirubin level should raise the index of suspicion ^30^. Other novel methods, including real-time polymerase chain reaction, have been explored but currently do not appear to be adequately sensitive to be used in this setting ^31^.

### Treatment of catheter-related bloodstream infection

Maintaining long-term central venous access is of prime importance in type 3 IF such that attempts to salvage an infected CVC are vital, wherever possible. Clearly, in a patient presenting with a suspected CRBSI and in septic shock, immediate blood cultures followed rapidly by CVC removal may be life-saving. Against usual microbiological despondency recommending routine CVC removal in all patients, two recent papers—from the UK ^21^ and the US ^32^—report success in the management of suspected CRBSIs in clinically stable patients with high salvage rates (72.5% and 70%, respectively) with antibiotics and, importantly, low mortality. Both the most commonly isolated and successfully treated microbe was coagulase-negative staphylococci (77.8–79.8%). High salvage rates were also observed with Gram-negative bacteria and poly-microbial infections. Traditionally, fungal CRBSIs are not salvaged, but the US team did demonstrate a very low success rate of 14.2% (6 out of 42) ^32^. There is still some debate surrounding *Staphylococcus aureus*, but both groups demonstrated success with this species.

### Prevention of catheter-related bloodstream infection

The reported incidence of CRBSI varies from 0.38 to 11.5 per 1,000 catheter days ^33, 34^. Although such variation may certainly relate to varied methods of diagnosis, there is no doubt that the extreme ranges of CRBSI rates result from varied practice in catheter care, particularly around education and training. As patients with IF are so reliant on the preservation of their venous access, the prevention of infections has to be a high priority amongst IF staff and patients, aided by strict protocolised measures for safe connection and disconnection. There is debate as to what impacts the risk of developing a CRBSI, but the number of infusion days ^35^, who manipulates the CVC ^35, 36^, and the type of CVC used ^37^ are likely important. Simple measures to reduce CRBSI rates include handwashing ^12, 38^ and 2% chlorhexidine cleaning ^25, 39, 40^. Ethanol locks have been shown to be very effective at reducing CRBSI rates (pre-introduction rates of 4.7–10.2 per 1,000 and post-introduction of 0.36–0.9 per 1,000 ^41– 43^) but because of the increased risk of catheter occlusion are not currently recommended by the ESPEN ^12, 44^. An alternative lock is taurolidine, an antimicrobial derived from the amino acid taurine, and data from open-label studies ^45^, retrospective treatment review ^32, 46, 47]^, and a small randomised controlled trial ^48^ demonstrate better outcomes. A meta-analysis of six randomised controlled trials reporting over 80,000 catheter days from 431 patients acknowledged methodological deficiencies in studies but reported that taurolidine locks compared with heparin locks reduced CRBSI risk by a risk ratio of 0.34 (95% confidence interval 0.21–0.55) without an increase in catheter occlusion (risk ratio 1.99, confidence interval 0.75–5.28) ^49^. In a randomised, double-blind, placebo-controlled trial, 41 high-risk patients with a CRBSI rate of 2.4 per 1,000 were assigned to taurolidine-citrate-heparin lock versus heparin only for 9,622 and 6,956 days, respectively. No CRBSI occurred in the taurolidine-citrate-heparin group compared with 7 CRBSI or 1.0 per 1,000 in the heparin group with associated reduction in CVC removal rate and cost of treatment for a CRBSI ^50^.

### Other central venous catheter complications

CVC infections are not limited to the intraluminal or the venous side of a CVC. Infections can also occur in the tunnel portion from where the CVC inserts into the skin until it passes into the vein; such tunnel infections have been reported to occur at a rate of 0.24–0.48 per 1,000 catheter days ^27, 51, 52^. Treatment usually requires systemic antibiotics but might also require CVC removal. Mechanical CVC complications occur and can be due to occlusion of the CVC or through kinks formed on particular movement or through the CVC falling out. Typically, occlusion occurs at a rate of 0.13–0.49 per 1,000 catheter days ^36, 51, 53– 57^.

CVC-related venous thrombosis (CRVT) occurred in large series at a rate of 0.08–0.2 per 1,000 catheter days ^58– 61^, but up to 2 out of 3 may go undetected ^61^. Whilst the aetiology is often unclear, endothelial damage by insertion of the catheter, flow dynamics within the vein, and a hypercoagulable state, including underlying clotting disorders, increase the risk, but, in addition, choline deficiency ^62^ and, as before stated, ethanol locks ^63^ may increase the risk of CRVT. There is currently no good evidence for primary prophylaxis to prevent a CRVT due to the risk of bleeding ^12^. However, once CRVT is diagnosed, treatment with low-molecular-weight heparin or oral anticoagulants, tailored to the patient’s circumstances (for example, absorption ability ^64^), is usually sufficient and this may need to be life-long ^12^. Although loss of venous access is an indication for intestinal transplantation (ITx) ^65^, it is not a common indication. To prevent progression of loss of venous access, more advanced treatment techniques are needed. Thrombolysis is indicated if the CRVT is diagnosed early, with a low risk of bleeding ^66^, but recurrence is high despite initial good resolution and continuing anticoagulation ^67^. With more mature thrombi, fragmentation or thrombectomy may be required to remove the clot and matured occluded veins may need venous recanalization ^68^. Venous stenosis following CRVT may require balloon angioplasty or stenting but also vascular grafts or bypass procedures. All of these novel techniques carry their own risks of morbidity and mortality and need appropriate discussion with the patient and the wider MDT.

## Other complications

### Intestinal failure-associated liver disease

IFALD is a common complication in children (estimates are 25–50% ^69^) but is far less common in adults, and severe liver dysfunction is observed in less than 5% of patients ^70, 71^. The pathophysiology of IFALD is multifactorial and includes intrahepatic inflammation associated with steatohepatitis, sepsis, nutrient deficiencies (choline, taurine, and essential fatty acids), nutrient excesses (lipid, glucose, and protein), dysfunctional biliary system (gall stones and bile acidification), medications, bacterial overgrowth, and other PN components, including plant phytosterols, exposure of PN to sunlight causing hydrogen peroxide production, heavy metals (aluminium, copper, and manganese), and use of polyvinyl chloride (PVC)-containing giving sets ^20, 72^. Maintaining enteral intake; replacing missing nutrients; administering cyclical PN; minimising calorie intake with lipids in particular; having lipid-containing mono-unsaturated fatty acids, fish oils, and a mixture of medium- and long-chain triglycerides; and preventing septic episodes, particularly CRBSI, are all thought to aid in protecting the liver ^20^. ****A common misconception is the suggestion that IFALD is similar to non-alcoholic fatty liver disease (NAFLD), but a very helpful review by Buchman *et al*. ^73^ has detailed the clear differences in clinical, biochemical, histological, and pathophysiological parameters between IFALD and NAFLD. The histological differences are recorded in Table 2, in which very clear patterns are observed in both IFALD and NAFLD ^73^. Where confusion comes, though, is in those patients who develop IF and whose body mass index does not fit the typical patient with IF ^74^. As the international obesity trend continues, there are more patients who will develop more of an overlap picture, especially if they have an antecedal metabolic syndrome, particularly those who have required bariatric surgery and develop subsequent IF ^75, 76^.

**Table 2.** Histological differences observed between intestinal failure-associated liver disease and non-alcoholic fatty liver disease.

Condition	Intestinal failure-associated liver disease	Non-alcoholic fatty liver disease
Cholestasis	Yes	No
Steatosis type	Macrovesicular and microvesicular	Mainly macrovesicular
Steatosis location	Periportal area	Pericentral area
Biliary tree changes	Obstruction: portal inflammation, oedema, ductal proliferation Ductopaenia	No obstruction No ductopaenia
Steatohepatitis	Rare	Common
Fibrosis	“Jigsaw” pattern: commences at portal end, then periportal followed by portal-portal bridging fibrosis then cirrhosis	Sinusoidal; ballooned hepatocytes with Mallory-Denk bodies; typically starts in pericentral area

### Metabolic bone disease

The combination of vitamin D deficiency, the underlying condition (for example, Crohn’s disease), and the mishandling of calcium and phosphate leads to metabolic bone disorders and 41–46% of patients on HPN develop osteoporosis on bone densitometry ^77, 78^.

### Renal disease

Renal dysfunction due to chronic dehydration is a recognised problem, but patients are also at risk of nephrolithiasis. This is in part due to dehydration, but, in particular, patients with jejunuo-colic anastomosis are at higher risk of oxalate nephropathy due to fatty acids preferentially binding calcium in the small bowel, leaving oxalate free to be absorbed and precipitate in the renal tubules ^79^.

### Quality of life and patient-related outcome measures

In children, QoL is comparable to that of healthy peers unless they have abdominal pain, and parental stress is reduced by an increased time from last operation or hospitalisation and increased by abdominal pain and a stool frequency of more than three per day ^80^. Adults report that HPN is a “lifeline” and that QoL is “good” to “wonderful”, and investigators found a strong desire for normalisation of life on HPN ^81^.

Until recently, patient-related outcome measures (PROMs) have typically been defined according to being on PN or nutritional autonomy, being employed or not, or survival. However, there is increasing evidence of a need for better understanding of PROMs, and research is under way to answer that question. The collection of QoL data has been mainly through the HPN-QoL devised by Baxter *et al*. ^82^. This comprehensive 48-point questionnaire covers so many areas of living with IF that it results in a wealth of data. In addition, the modified ITx-QOL by Pironi and Baxter changes a few key questions to try to match the flow of patient experience across HPN and ITx ^26^. However, many patients find 48 questions quite daunting, and, notably, such health-related QoL questionnaires have principally focussed on impairments and functional limitations that are of interest to health professionals. A recent PROM has been developed (Parenteral Nutrition Impact Questionnaire [PNIQ]) that differs from previous tools, as its items were generated from unstructured qualitative interviews conducted with patients and therefore should provide a more patient-centric assessment of QoL ^83^. Moreover, PNIQ provides a unidimensional index of QoL through only 20 dichotomous items that can be completed within minutes, making it a useful tool for day-to-day clinical practice and also to evaluate the impact of novel CIF therapies on QoL.

## Evolving strategies for managing intestinal failure

### Growth factors

Many different hormonal treatments have been tried to treat SBS by enhancing intestinal adaptation and absorption and reducing PN requirements in CIF, although frequently the effects are not sustained and patients revert to pretreatment states relatively quickly. Typical treatments include growth hormone ^84^ and glucagon-like peptide-1 (GLP-1) and GLP-2 agonists. The recombinant analogue GLP-2 teduglutide (Shire Pharmaceuticals) was approved in 2012 for the treatment of adults with SBS dependent on PN despite optimal medical therapy. It has been shown to reduce PN requirements in large randomised placebo-controlled studies with open-label extensions ^85– 87^. Teduglutide in children over a 12-week study has also demonstrated benefit with reduction of PN volume and calories by 25–41% and 45–50%, respectively, in higher doses of teduglutide (0.025 and 0.05 mg/kg per day) compared with 0% and 1–6% for low dose (0.0125 mg/kg per day) and standard of care; there was a compensatory rise in enteral nutrition volume of 22–40% in teduglutide groups compared with 11% for standard treatment. Four patients achieved independence from PN ^88^. GLP-1 analogues may also offer promise of restoring gut function by applying a gastric emptying brake as noted in an 8-week pilot study of eight patients with SBS given once-daily liraglutide (Novo Nordisk) ^89^. A further pilot study demonstrated enhanced absorption with combination GLP-1 and GLP-2 analogues ^90^.

### Intestinal lengthening

Three different intestinal lengthening procedures exist: the Bianchi procedure ^91^, a longitudinal incision of a dilated segment of small bowel to then place two new sections end to end; the serial transverse enteroplasty (STEP) ^92^, diagonal non-occlusive breaks in the dilated small bowel to form an increased length and slower transit time; and the spiral intestinal lengthening and tailoring (SILT) ^93^, a spiral incision around the dilated small bowel then resewn to tailor the length. The majority of articles reporting intestinal lengthening are paediatric reports. One of the largest adult series ^94^ demonstrated, in 20 adults (six Bianchi and 15 STEP; one adult had two procedures) over a 15-year period, that the mean length of gut increased from 60 cm pre-surgery to 80 cm post-surgery and, in 17, 10 (59%) had weaned off PN, and seven achieved increased enteral calorie intake. For the other three, two patients died during the follow-up period and one required an intestinal transplant. A recent systematic review and meta-analysis of STEP on enteral tolerance in children included seven case series of 86 children and found that 16 (19%) out of 86 children had no improvement, but there was an increase in enteral tolerance from 35% to 70% following STEP. They gave a summary estimate improvement in 87% of children following STEP ^95^.

### Intestinal transplant

Intestinal transplant is a critically important salvage operation to replace lost or diseased gut to prevent the progression of IFALD, to prevent further complications associated with long-term HPN (particularly loss of venous access), and to increase longevity in those with increased risk of death due to IF, such as patients with intra-abdominal desmoids ^65, 96^. The grafts include liver-containing grafts (liver-small bowel or multivisceral) or liver-free grafts (isolated small bowel including small bowel and colon or modified multivisceral including stomach, pancreas, small bowel, and colon). Patient survival rates continue to improve, and the last international registry data reported 1-, 5-, and 10-year survival as 76%, 56%, and 43%, respectively ^97^. Yet complications of rejection, graft-versus-host disease, post-transplant lymphoproliferative disorder, and infection still cause constant vigilance for patients and transplant units. Currently, ITx remains a rescue procedure in the face of life-threatening complications owing to the superior survival for patients with HPN. However, as life expectancy following transplantation improves, the balance and timing of transplantation may shift earlier in the disease course as observed in other solid organ transplantation.

## Conclusions

IF is an uncommon condition, but the impact on the individual is enormous. The MDT approach to facilitate safe care is paramount to ensuring longevity in these complex patients. In addition, there appears to be a necessity to improve outcomes where prevention is by far better than cure for multiple complications of IF, including CRBSI, venous thrombosis, and IFALD. As growth factors develop, opportunities to wean patients off PN become closer to reality. In the absence of relatively cheap hormonal treatments to facilitate intestinal adaption, surgical rescue procedures are vital to maintain functioning gut: first, as much gut as possible is brought into circuit and then intestinal lengthening and transplant remain important options. Finally, and perhaps most importantly, to reduce many of the surgical catastrophes that ultimately result in patients developing IF, education initiatives and IF centres of excellence play a vital role.
